# TALEN mediated targeted mutagenesis of the *caffeic acid O-methyltransferase* in highly polyploid sugarcane improves cell wall composition for production of bioethanol

**DOI:** 10.1007/s11103-016-0499-y

**Published:** 2016-06-15

**Authors:** Je Hyeong Jung, Fredy Altpeter

**Affiliations:** 1 Agronomy Department, University of Florida, IFAS, PO Box 110300, Gainesville, FL 32611 USA; 2 Plant Molecular and Cellular Biology Program, University of Florida, IFAS, PO Box 110300, Gainesville, FL 32611 USA; 3 Agronomy Department, University of Florida-IFAS, PO Box 103610, Gainesville, FL 32611 USA; 4 Institute of Life Science and Natural Resources, Korea University, 145 Anam-ro, Seongbuk-gu, Seoul, 02841 Republic of Korea

**Keywords:** Transcription activator-like effector nucleases, Genome editing, *Caffeic acid O-methyltransferase*, Lignin, Sugarcane, Capillary electrophoresis

## Abstract

Sugarcane (*Saccharum* spp. hybrids) is a prime crop for commercial biofuel production. Advanced conversion technology utilizes both, sucrose accumulating in sugarcane stems as well as cell wall bound sugars for commercial ethanol production. Reduction of lignin content significantly improves the conversion of lignocellulosic biomass into ethanol. Conventional mutagenesis is not expected to confer reduction in lignin content in sugarcane due to its high polyploidy (*x* = 10–13) and functional redundancy among homo(eo)logs. Here we deploy transcription activator-like effector nuclease (TALEN) to induce mutations in a highly conserved region of the *caffeic acid O-methyltransferase* (*COMT*) of sugarcane. Capillary electrophoresis (CE) was validated by pyrosequencing as reliable and inexpensive high throughput method for identification and quantitative characterization of TALEN mediated mutations. Targeted *COMT* mutations were identified by CE in up to 74 % of the lines. In different events 8–99 % of the wild type *COMT* were converted to mutant *COMT* as revealed by pyrosequencing. Mutation frequencies among mutant lines were positively correlated to lignin reduction. Events with a mutation frequency of 99 % displayed a 29–32 % reduction of the lignin content compared to non-transgenic controls along with significantly reduced S subunit content and elevated hemicellulose content. CE analysis displayed similar peak patterns between primary *COMT* mutants and their vegetative progenies suggesting that TALEN mediated mutations were faithfully transmitted to vegetative progenies. This is the first report on genome editing in sugarcane. The findings demonstrate that targeted mutagenesis can improve cell wall characteristics for production of lignocellulosic ethanol in crops with highly complex genomes.

**Electronic supplementary material:**

The online version of this article (doi:10.1007/s11103-016-0499-y) contains supplementary material, which is available to authorized users.

## Introduction

Sugarcane (*Saccharum* spp. hybrids) is currently the most efficient crop for bioethanol production due to its exceptional biomass yield, perennial growth and the accumulation of fermentable sugars in stem internodes. The first generation process for ethanol production from sugarcane consists of sugarcane cleaning and preparation, extraction of sugars from stems, juice treatment, concentration and fermentation, distillation, dehydration and cogeneration, as described by Junqueira et al. (2011). An abundant amount of lignocellulosic sugarcane biomass residues (bagasse, straw and tops) are generated during the production of ethanol or sugar. Utilizing cell wall bound sugars from lignocellulosic sugarcane biomass requires second generation conversion technologies. Integration of first and second generation technologies for commercial ethanol production is already taking place in Brazil (Dias et al. 2014). This will drastically boost the biofuel yield per ton of harvested sugarcane and also improve the sustainability of the process (Dias et al. 2014).

Presence of lignin in plant cell wall significantly limits bioconversion of lignocellulosic biomass into biofuels (Jørgensen et al. 2007; Zhao et al. 2012). Improvement of saccharification efficiencies and biofuel yields have been demonstrated in a number of feedstock plants by reducing their lignin content (Chen and Dixon 2007; Saballos et al. 2008; Fu et al. 2011; Jung et al. 2013). Isolation of mutants or RNA interference (RNAi) mediated suppression of lignin biosynthetic gene(s) have been employed to create feedstock plants more amenable to biofuel conversion. However, for sugarcane conventional mutagenesis is not expected to confer reduction in lignin content due to its high polyploidy and functional redundancy among homo(eo)logs. Alternatively, RNAi suppression of one of the lignin biosynthetic genes has been successfully applied to sugarcane and reduced lignin content by up to 12 % (Jung et al. 2012, 2013). Although RNAi is a straightforward approach, which can target a specific gene and its homo(eo)logs simultaneously, this approach requires that the RNAi inducing transgene construct is stably expressed. Thus, targeted mutagenesis of a gene could be more stable compared to RNAi mediated suppression.

Modern sugarcane cultivars are highly polyploid (*x* = 10–13) and frequently aneuploid interspecific hybrids between *Saccharum officinarum* and *Saccharum spontaneum* with large number of chromosomes (100–130) and genome size (~10 Gb) (Le Cunff et al. 2008; de Setta et al. 2014). During the past century, sugarcane yields have been improved dramatically through tremendous efforts in breeding combined with better agronomic practices. However, its complex genome structure and poor fertility made crop improvement through conventional and marker-assisted breeding very challenging (Jackson 2005; Dal-Bianco et al. 2012). Genetic engineering allows stacking of superior alleles into elite sugarcane cultivars without the need to screen thousands of segregating progenies. Recently developed sequence-specific nucleases (SSNs) will allow precise and targeted modifications of the complex sugarcane genome. These SSNs currently include zinc-finger nucleases (ZFN) (Kim et al. 1996), meganucleases (Smith et al. 2006), transcription activator-like effector nucleases (TALEN) (Christian et al. 2010; Morbitzer et al. 2010), and RNA guided nuclease (CRISPR/Cas) systems (Jinek et al. 2012). These technologies combine customizable sequence specific DNA binding proteins (or guide RNA) with a non-specific nuclease domain to create double strand breaks at target sites of interest. Targeted mutagenesis can be induced at the cleaved sites by non-homologous end joining (NHEJ) in absence of a template DNA. Homology directed repair (HDR) mechanisms, can facilitate DNA insertion or sequence replacement in the presence of repair/donor template. SSNs have proven their capability in genome editing in various organisms including a number of plant species (Kim and Kim 2014; Voytas 2013). More recently, these tools have been applied to crop species to improve agronomic traits, such as disease resistance in rice and wheat, phytase activity in barley, oil quality in soybean, fragrance in rice, cold storage and processing traits in potato (Li et al. 2012; Wendt et al. 2013; Haun et al. 2014; Wang et al. 2014; Shan et al. 2015; Clasen et al. 2016). Here, we report TALEN mediated targeted mutagenesis of the lignin biosynthetic gene, *caffeic acid O-methyltransferase* (*COMT*) to reduce the lignin content in sugarcane.

## Results

### Generation of TALEN induced *COMT* mutant lines

TALEN binding sites were selected in the conserved region of the first exon among the sugarcane *COMT* gene and its putative homo(eo)logs. TALEN arms were custom synthesized to target the selected region of *COMT*. The length of TALEN binding sites and spacer were 16 and 17 bp, respectively. The location and sequence of TALEN binding sites, and the corresponding repeat variable di-residues (RVDs) of each TALEN arm are shown in Supplemental Fig. S1. The cleavage rate of TALEN was 95 % based on the yeast single strand annealing assay (Supplemental Fig. S2).

TALEN expression cassette was introduced into sugarcane by *Agrobacterium* mediated transformation via indirect embryogenesis (AIE) or biolistic mediated transformation via direct embryogenesis (BDE). TALEN integration was evaluated by PCR in the regenerated plants, and a total of 39 and 27 TALEN integrated lines were generated by AIE and BDE, respectively (data not shown).

**Fig. 1:**
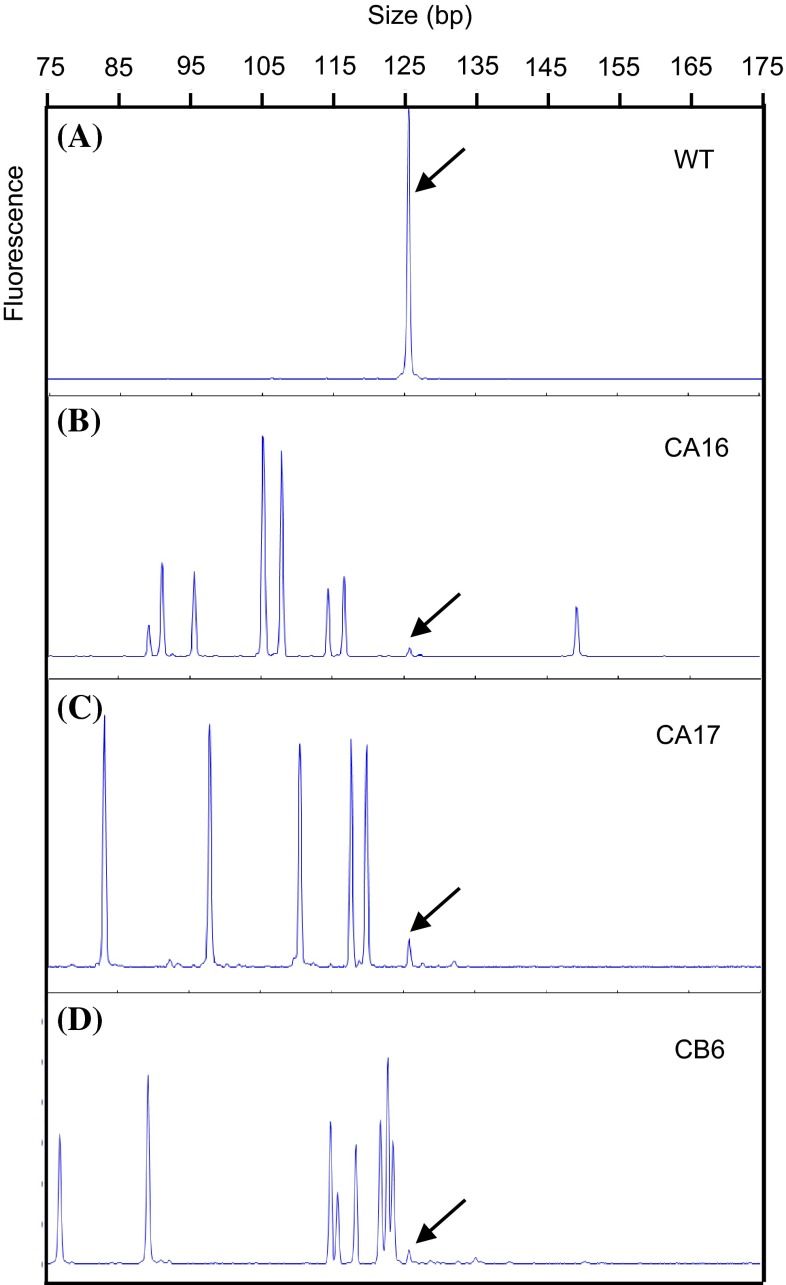
Selection of TALEN mediated *COMT* mutants using capillary electrophoresis (CE). Electropherogram of *COMT* amplicon encompassing the TALEN target site from wild type (WT) (**a**) and representative mutant lines (CA17, CA16, and CB6) (**b–d**) are displayed. *Arrows* indicate the peaks from the wild type *COMT* amplicon at 125 bp. Peaks shorter or longer than 125 bp represent deletions or insertions at TALEN target sites

TALEN induced insertions and/or deletions (indels) at the target site were identified by capillary electrophoresis (CE) of the *COMT* amplicon encompassing the TALEN target site (Fig. 1). *COMT* amplicon from wild type sugarcane was represented as one single peak at 125 bp in the CE electropherogram (Fig. 1a), while the amplicons from mutant lines showed size variations indicating indels at the target site (Fig. 1b–d). All of the mutant lines had multiple peaks indicating the presence of multiple mutant types, and a peak from wild type *COMT* was also detected at 125 bp in all of the mutant lines. The number of mutant lines generated by AIE and BDE was 29 and 8 lines, respectively. The efficiency of TALEN mediated mutagenesis (number of mutant lines over lines with integration of TALEN into the sugarcane genome) was 29/39 (74 %) and 8/39 (30 %) for AIE and BDE, respectively.

### Sequence analysis of TALEN mediated *COMT* mutant lines

**Fig. 2:**
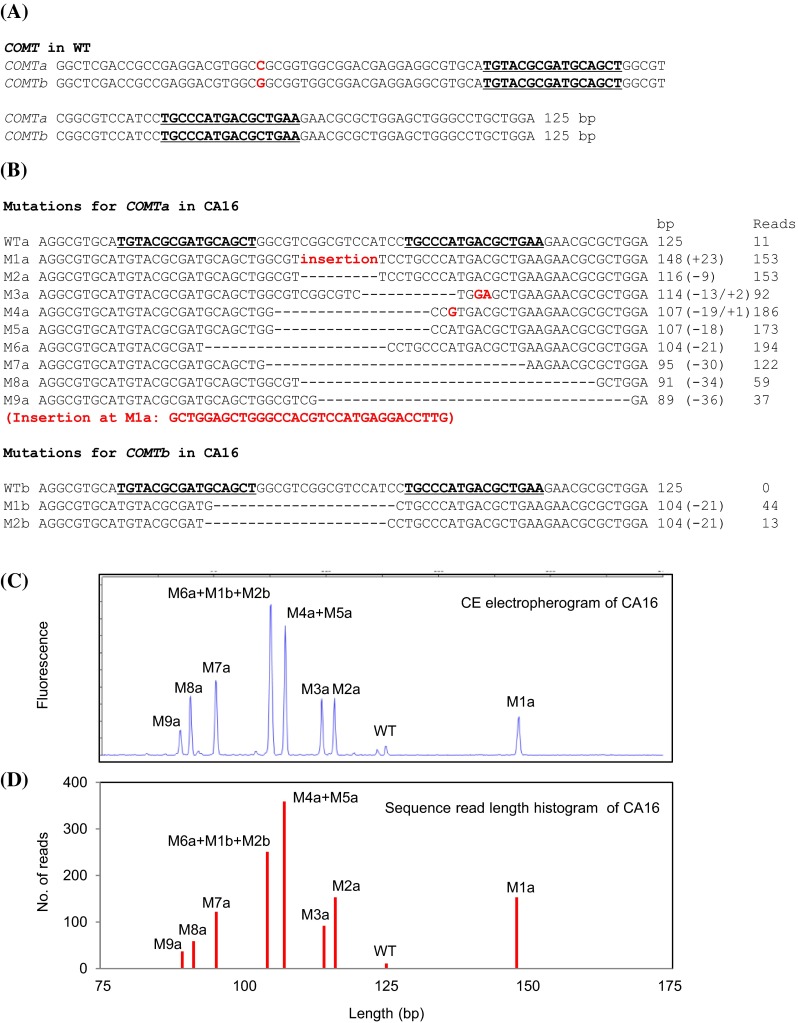
TALEN mediated *COMT* mutations. 125 bp of amplicon encompassing TALEN target site was sequenced by 454 pyrosequencing in wild type (WT) and the selected mutant lines. TALEN binding sequences are underlined and shown in* bold* characters. **a** Sequence comparison between two putative *COMT* homo(eo)logs (*COMTa* and *COMTb*) in WT. A nucleotide indicated in *red color* indicates single nucleotide polymorphism (SNP) between *COMTa* and *COMTb*, which is located outside of TALEN target sites. **b** Sequences of TALEN induced *COMT* mutants for *COMTa* and *COMTb* in one of the mutant lines, CA16. *WTa* and* WTb* wild type *COMTa* and *COMTb, M1a*–*M9a* nine different mutants for *COMTa, M1b and M2b* two different mutants for *COMTb*. Insertions or substitutions are indicated by *red letters*. Deletions are indicated by *dashes*. The numbers below ‘bp’ are the length of the sequence, and numbers with + or − indicate the size of the insertions (substitutions) or deletions, respectively. The numbers below ‘reads’ are the number of sequence reads for each WT and mutant type. **c, d** CE electropherogram and sequence read length histogram from the mutant line, CA16. Each mutant type (M1a–M9a for *COMTa* and M1b, M2b for *COMTb*) identified by sequencing corresponds to each peak from CE electropherogram

454 pyrosequencing of *COMT* amplicon encompassing the TALEN target site was performed in selected mutant lines to confirm TALEN induced *COMT* mutations and validate CE results. Sequencing data revealed the presence of at least two putative *COMT* homo(eo)logs (*COMTa* and *COMTb*) displaying one nucleotide sequence difference within the sequenced region (Fig. 2a). In wild type, from a total 3884 reads, the vast majority of reads (3735, 96 %) originated from *COMTa*, and 149 reads (4 %) were from *COMTb* (Table 1).

**Table 1:** The number of mutant types, sequence reads, and mutation frequencies in TALEN induced *COMT* mutant lines

Lines	No. of mutant types (*COMTa, COMTb*)	No. of total sequence reads (*COMTa, COMTb*)	No. of mutant sequence reads (*COMTa, COMTb*)	No. of WT sequence reads (*COMTa, COMTb*)	Mutation frequency (%)	
					SEQ	RF
WT	0	3884 (3735, 149)	0	3884 (3735, 149)	0	0
CA1	13 (12, 1)	1360 (1313, 47)	1307 (1261, 46)	53 (52, 1)	96.1	96.6
CA13	13 (12, 1)	1419 (1392, 27)	1405 (1378, 27)	14 (14, 0)	99.0	98.0
CA14	11 (10, 1)	1256 (1204, 52)	211 (204, 7)	1045 (1000, 45)	16.8	16.2
CA16	11 (9, 2)	1237 (1180, 57)	1226 (1169, 57)	11 (11, 0)	99.1	98.5
CA17	16 (13, 3)	1868 (1841, 27)	1833 (1808, 25)	35 (33, 2)	98.1	96.7
CA25	9 (8, 1)	673 (632, 41)	653 (612, 41)	20 (20, 0)	97.0	93.7
CB1	5 (5, 0)	3433 (3394, 39)	686 (686, 0)	2747 (2708, 39)	20.0	27.0
CB2	2 (2, 0)	730 (715, 15)	57 (57, 0)	673 (658, 15)	7.8	16.4
CB3	55 (45, 10)	1573 (1534, 39)	1433 (1396, 37)	140 (138, 2)	91.1	90.3
CB5	69 (63, 6)	1277 (1243, 34)	1177 (1144, 33)	100 (99, 1)	92.2	87.0
CB6	8 (7, 1)	550 (542, 8)	516 (511, 5)	34 (31, 3)	93.8	98.8
CB7	75 (66, 9)	1549 (1494, 55)	1395 (1346, 49)	154 (148, 6)	90.0	85.3
CB8	83 (78, 5)	1526 (1501, 25)	1386 (1363, 23)	140 (138, 2)	90.8	83.4

Numbers inside of parentheses are the values for COMTa and COMTb, respectively

*SEQ* mutation frequency estimated by the number of sequence reads. Mutation frequency (%) = (The number of mutant sequence reads over the number of total sequence reads) × 100

*RF* mutation frequency estimated by relative fluorescent quantitation based on CE electropherogram. Mutation frequency (%) = (Sum of peak height of all mutant peaks over sum of peak height of all peaks including wild type peak) × 100

*WT* wild type sugarcane, *CA1*–*CA25* TALEN induced mutant lines generated by AIE, *CB1*–*CB8* TALEN induced mutant lines generated by BDE

All of the analyzed mutant lines were confirmed to carry mutations at the TALEN target sites. An example of mutant *COMT* sequences in one of the mutant lines, CA16 is shown in Fig. 2b. A total of 11 different mutant types (nine for *COMTa* and two for *COMTb*) were observed in CA16. The mutant types, M3a, M8a, M9a for *COMTa* and M2b for *COMTb* were relatively infrequent displaying lower sequence read numbers than other mutant types. Among 11 mutant types found in CA16, ten were deletions ranging from 9 to 36 bp, and one mutation (M1a) was a 23 bp insertion (Fig. 2b). One to two nucleotide substitutions (M3a and M4a) were also observed (Fig. 2b).

Multiple mutant types were detected in all of the analyzed mutant lines and the number of mutant types differed in different mutant lines (Table 1). The number of mutant types for *COMTa* and *COMTb* ranged from 2 to 78 and 0 to 10, respectively. The majority of mutant lines displayed less than 13 mutant types for *COMTa* and less than 3 mutant types for *COMTb* (Table 1). Four mutant lines all derived from BDE had relatively large number of mutant types (45–78 for *COMTa* and 6–10 for *COMTb*). Of 13 analyzed mutant lines, 11 mutant lines displayed simultaneous TALEN induced mutations in both of the *COMTa* and *COMTb*.

Among a total of 370 mutant types found in analyzed mutant lines, the majority of the mutations were deletions (98 %), and the length of deletions was in the range from 1 to 52 bp. The most frequent mutation was 5 bp deletion (10 %) followed by 6 and 7 bp deletions. Insertions were relatively infrequent (2 %), and the length of insertions was between 2 and 23 bp (Supplemental Fig. S3).

Mutation frequency in the amplicon population of *COMT* was estimated based on the ratio of the number of mutant sequence reads to the number of total sequence reads including wild type sequence reads. With more than 1000 sequence reads per line, wild type *COMT* sequence was detected in all of the analyzed mutant lines, with the lowest frequency in line CA16 (0.9 % of all reads) and the highest frequency in line CB2 (92.2 % of all reads) (Table 1). Accordingly, the mutation frequencies ranged from 7.8 to 99.1 % among the analyzed mutant lines. Of 13 analyzed lines, nine lines had mutation frequencies over 90.0 %.

Estimation of mutation frequency by CE based on relative fluorescence of mutant and wild type peak gave similar results as the confirmation of mutation frequency by 454 sequencing (Table 1). Each mutant type identified by sequencing with different length was represented by a peak with corresponding length on CE electropherogram (Fig. 2b, c). Peak intensity from CE electropherogram corresponded well to the number of sequence reads (Fig. 2c, d).

### Cell wall characteristics and phenotype in TALEN mediated *COMT* mutant lines

Total lignin content was evaluated from control plants (WT, TCA, and TCB), and mutant lines (Table 2). There was no difference in lignin content among control plants. The lines (CA14, CB1, and CB2) with relatively low mutation frequencies (7.8–20.0 %) did not show a reduction in lignin content. Lignin reduction was positively correlated with mutation frequencies among mutant lines with mutation frequencies between 90 and 99 % (R^2^ = 0.85, Supplemental Fig. S4). Lignin reduction levels ranged from 11 to 32 % in the mutation lines with relatively high mutation frequencies (90–99 %) compared to wild type (WT). All of the lignin reduced *COMT* mutant lines showed significantly reduced S subunit content by up to 54 %, while G subunit content was unchanged compared to WT (Supplemental Table 1).

**Table 2:** Total lignin content and phenotype in TALEN induced *COMT* mutant lines

Lines	Mutation frequency (%)^a^	Total lignin^b^ (mg/g DW)	Reduction^c^ (%)	Stem diameter (mm)^d^	Brown coloration	
					Internode	Midrib
WT1	0	250.2 ± 13.2	–	20.7	N	N
WT2	0	252.2 ± 4.7	–	19.9	N	N
TCA	0	251.6 ± 1.4	–	17.6*	N	N
TCB	0	257.3 ± 5.9	–	19.6	N	N
CA1	96.1	178.3* ± 28.7	30	16.3*	B	B
CA13	99.0	173.1* ± 9.8	32	15.2*	B	B
CA14	16. 8	262.9 ± 7.1	−4	17.9*	N	N
CA16	99.1	179.8* ± 10.4	29	16.3*	B	B
CA17	98.1	193.4* ± 11.2	24	16.6*	B	B
CA25	97.0	191.2* ± 9.6	24	16.1*	B	B
CB1	20.0	247.7 ± 3.4	2	20.4	N	N
CB2	7.8	240.5 ± 17.6	5	20.4	N	N
CB3	91.1	214.3* ± 12.7	15	19.6	B	N
CB5	92.2	223.0* ± 12.7	12	20	B	N
CB6	93.8	197.2* ± 14.2	22	21.4	B	B
CB7	90.0	224.1* ± 15.5	11	21.1	B	N
CB8	90.8	220.3* ± 9.4	13	19.3	B	N

*WT1 and WT2* wild type sugarcane, *TCA* transgenic control plant generated by AIE containing only *npt*II gene, *TCB* non-mutated transgenic control plant generated by BDE harboring TALEN expression cassette, *CA1*–*CA25* TALEN induced mutant lines generated by AIE, *CB1*–*CB8* TALEN induced mutant lines generated by BDE, *N* no brown coloration observed, *B* brown coloration observed, *DW* dry weight

^a^Mutation frequencies were estimated by the number of sequence reads

^b^Values are means ± standard deviation. *Asterisk* indicates a significant difference in total lignin content between WT1 and a mutant line (*n* = 2, *P* < 0.05 in *t* test)

^c^Reduction levels of lignin content in mutant lines are compared with the mean of lignin contents among control plants (WT1, WT2, TCA, and TCB)

^d^Values are means of stem diameter from two different tillers of mutant lines. *Asterisk* indicates a significant difference in stem diameter compared with WT1 (*n* = 2, *P* < 0.05 in *t* test)

**Fig. 3:**
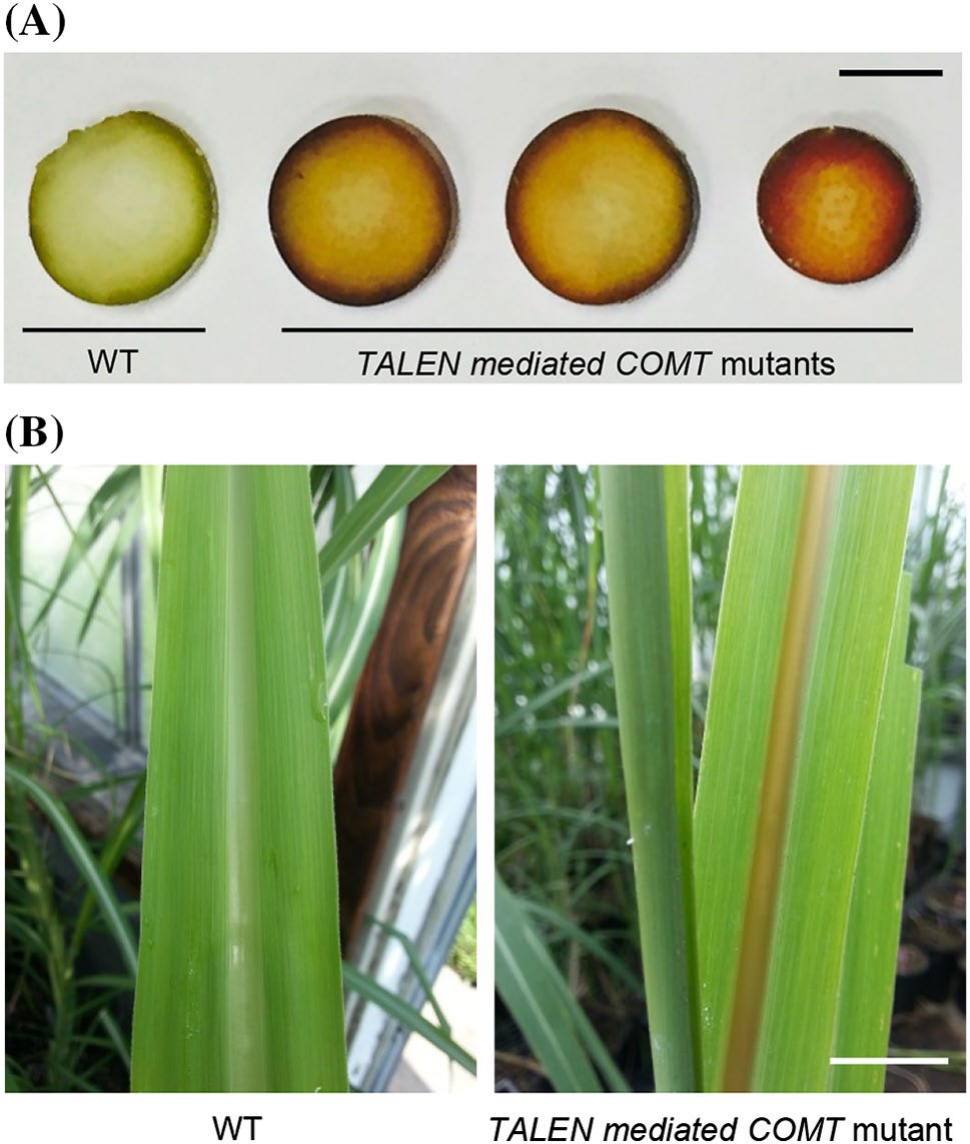
*COMT* mutant phenotype. **a** *Brown coloration* in internodes of *COMT* mutant plants. The basal internodes were transversely sectioned and the picture was taken immediately without fixation and staining. **b** *Brown coloration* in the midribs of immature leaves of *COMT* mutants. *WT* wild type. *Scale bars* indicate 1 cm

Stem diameter was measured from two different tillers of WT, transgenic controls generated by AIE (TCA) and BDE (TCB), and primary mutant lines (Table 2). Lignin reduced mutant lines generated by BDE showed comparable stem diameter with WT. Stem diameters of TCB and mutants without lignin reduction (CB1 and CB2) were also similar with that of WT. Lignin reduced mutant lines generated by AIE showed 20–27 % reduction in stem diameter compared with WT, while corresponding control (TCA) and the mutant with no lignin reduction (CA14) also showed 14 and 15 % reduction in stem diameter, respectively. All of the lignin reduced mutant lines exhibited brown coloration in leaf midrib and/or internodes, while the lines (CA14, CB1, and CB2) with no lignin reduction did not show brown coloration (Fig. 3; Table 2). Of ten mutant lines showing lignin reduction, six lines (CB6, CA1, CA13, CA16, CA17, and CA25) with relatively high lignin reduction (22–32 %) displayed brown coloration in both of the internodes and midribs. Brown coloration in midribs was observed in immature leaves including leaf whorls and top visible dewlap leaf. Four lines with relatively low lignin reduction (11–15 %) did not show brown coloration in leaf midrib.

The effect of *COMT* mutation and lignin reduction on cell wall components, cellulose and hemicellulose, was investigated by analyzing cell wall carbohydrates (Table 3). The amount of glucose which is mostly derived from cellulose did not differ significantly between lignin reduced *COMT* mutant and WT. The amounts of xylose and arabinose, two major components of hemicellulose were significantly higher than WT in two mutant lines (CA16 and CA17) with high mutation frequency and lignin reduction levels (Tables 2, 3). Other lignin reduced mutant lines did not show significant difference in xylose and arabinose content compared to WT. The amount of soluble solids in the stalk did not differ significantly between mutant lines CB6, CA14 and WT, while some of the lines (CA16, CA17, CB5, and CB7) displayed a 17–20 % reduction (Table 3).

**Table 3:** Cell wall carbohydrates and soluble solids in TALEN induced *COMT* mutants and control plants

Lines	Cell wall carbohydrates (mg/g DW)^a^			Soluble solids brix^a^
	Glucose	Xylose	Arabinose	
WT	446.7 ± 4.9	198.7 ± 8.9	25.6 ± 1.3	21.5 ± 0.35
TCA	459.1 ± 6.6	209.2 ± 10.7	27.2 ± 0.0	20.9 ± 0.35
TCB	449.5 ± 3.2	190.7 ± 1.6	28.6 ± 1.6	20.9 ± 0.99
CA14	452.0 ± 11.4	212.0 ± 2.2	29.4 ± 0.4	20.7 ± 0.27
CA16	460.3 ± 12.1	228.1 ± 5.8*	31.3 ± 0.9*	17.4 ± 0.78*
CA17	455.9 ± 2.1	238.4 ± 0.7*	31.1 ± 0.7*	17.1 ± 1.03*
CB5	452.9 ± 1.1	216.3 ± 1.1	28.3 ± 0.7	17.9 ± 1.03*
CB6	462.9 ± 6.2	213.3 ± 5.8	27.2 ± 0.0	20.9 ± 0.53
CB7	455.1 ± 5.6	216.6 ± 6.0	29.2 ± 1.1	17.6 ± 0.27*

*WT* wild type sugarcane, *TCA* transgenic control plant generated by AIE containing only *npt*II gene, *TCB* non-mutated transgenic control plant generated by BDE harboring TALEN expression cassette, *CA14, CA16, and CA17* TALEN induced mutant lines generated by AIE, *CB5, CB6, and CB7* TALEN induced mutant lines generated by BDE, *DW* dry weight

^a^Values are means ± standard deviation from two different tillers of mutant lines. *Asterisk* indicates a significant difference in carbohydrate content, or soluble solids between WT and a mutant line (*n* = 2, *P* < 0.05 in *t* test)

### Transmission of TALEN mediated mutations in vegetative progenies

**Fig. 4:**
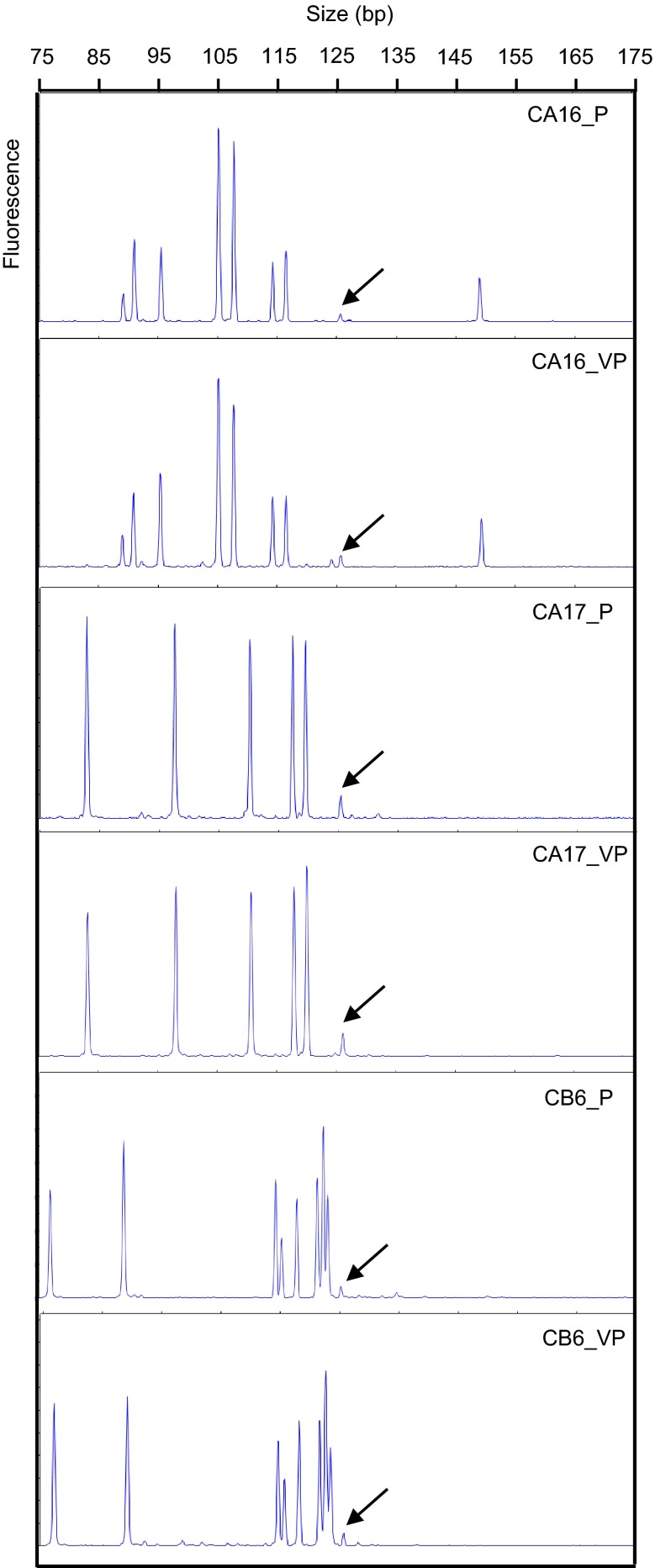
Transmission of TALEN mediated *COMT* mutation in vegetative progenies. CE electropherogram of *COMT* amplicon from primary mutant lines (CA16_P, CA17_P, and CB6_P) and their vegetative progenies (CA16_VP, CA17_VP, and CB6_VP). *Arrows* indicate the peaks from the wild type *COMT* amplicon at 125 bp

The selected mutant lines were clonally propagated by planting single-node segments of primary mutant plants. CE of *COMT* amplicon encompassing the TALEN target site was performed in two individual vegetative progenies to investigate transmission of TALEN induced mutations. CE electropherograms were comparable between primary mutants and their vegetative progenies suggesting that TALEN mediated mutations were transmitted to vegetative progenies (Fig. 4).

## Discussion

Combining many superior alleles in vegetatively propagated, elite sugarcane cultivars by traditional breeding is very challenging due to the frequently aneuploid and highly polyploid genome. Genome editing with SSN will be particularly beneficial for sugarcane improvement since this technology allows addition of superior alleles to elite cultivars without the need of repeated back crossing and screening of thousands of segregating progenies. Genome editing was deployed here for the first time for sugarcane improvement by using TALEN targeted to a conserved region of *COMT* and resulted in drastic reduction of the lignin content.

The conversion efficiency of lignocellulosic biomass into biofuel is negatively correlated with total lignin content in feedstock plants (Chen and Dixon 2007; Studer et al. 2011). Thus, reducing lignin content has been a straightforward strategy to improve biofuel yield from lignocellulosic biomass (Chen and Dixon 2007; Fu et al. 2011; Baxter et al. 2014). RNAi mediated suppression of the lignin biosynthetic gene *COMT* recently successfully reduced lignin content in sugarcane up to 12 % following 91 % suppression of transcripts. Saccharification efficiency correlated to lignin suppression levels and was increased by up to 32 % (Jung et al. 2012, 2013). However, RNAi mediated gene suppression, typically requires the generation of a large number of transgenic lines to identify an event with desired suppression levels, phenotype and agronomic performance. Further, the stability of the desired gene suppression level depends on continued transgene expression in subsequent generations, making this approach less predictable. In an attempt to overcome these limitations, we deployed TALEN mediated targeted mutagenesis of *COMT* in the commercially important sugarcane cultivar CP88-1762. In this study, TALEN mediated targeted *COMT* mutagenesis resulted in drastic reduction in lignin content by up to 32 % along with reduction of syringyl subunits by up to 54 %.

TALEN scaffolds and transformation system applied in this study enabled targeted *COMT* mutation with an efficiency of up to 74 %. This mutation rate compares very favorably with earlier reported TALEN mediated targeted mutagenesis ranging from 4 to 31 % (Shan et al. 2013; Wendt et al. 2013; Zhang et al. 2013, 2016). The observed high mutation rate can be a result of the TALEN scaffold, target genes, and target region. Our data also suggests that the tissue culture system and/or gene transfer method also affect the efficiency of TALEN mediated mutagenesis. Agrobacterium mediated gene transfer into embryogenic calli resulted in a 2.5 times higher mutation rate than biolistic gene transfer followed by direct embryogenesis. Chromatin de-condensation is a common feature in dedifferentiated cells (i.e. callus cells), and DNA in those cells is more accessible to endo-exonuclease than in differentiated cells (Blank et al. 1992; Grafi 2004; Exner and Hennig 2008). Therefore, the less condensed chromatin configuration in calli might promote the access of TALEN to the target region and increase the efficiency of nuclease activity. Similarly, Mikami et al. (2015) reported that extending the callus period increases the proportion of mutated cells with CRISPR/Cas9 mediated genome editing.

For rapid identification of SSN mediated genome editing events, several screening methods have been described in the literature, such as sequencing, melting curve analysis, or nuclease assays cutting heteroduplex dsDNA or restriction sites located in the SSN target site. However, these methods are rather expensive, laborious, or insensitive. CE of target gene amplicons was applied here for the first time for rapid identification of TALEN induced mutations. CE was validated by 454 pyrosequencing as reliable and inexpensive high throughput method for identification and quantitative characterization of TALEN mediated mutations. Amplicons encompassing the target site are simply prepared by PCR with primers labelled with fluorescent molecules, and analyzed in a high-throughput manner. CE is able to deliver additional genotyping data. In this study, CE allowed the identification of different mutant types and their frequencies in the amplicon population.

The presence of multiple mutant types could be the consequence of simultaneous and independent mutagenic events among homo(eo)alleles. TALEN mediated simultaneous mutagenesis of homo(eo)alleles or family members was recently described in soybean, hexaploid wheat, and potato (Haun et al. 2014; Wang et al. 2014; Clasen et al. 2016). The TALEN target site is located in the highly conserved first exon of the sugarcane *COMT* gene. Therefore, PCR amplicons encompassing the TALEN target site which were used for 454 sequencing or CE differentiate only between two of the *COMT* alleles. The exact number of *COMT* alleles/copies is not known in the highly polyploid sugarcane. However, both WT-peak intensity in CE and ratio of mutant to WT reads following 454 sequencing correspond, and the majority of analyzed sugarcane *COMT* mutants (nine out of 13 lines) exhibited a mutation frequency between 90 and 99 %. This high conversion rate of wild type to mutant loci suggests that the conservation of the chosen target site supports multiallelic knockouts of the homo(eo)logous *COMT*. CE electropherogram analysis displayed similar peak patterns between primary mutants and their vegetative progenies suggesting that TALEN mediated mutations were transmitted to vegetative progenies.

The majority of TALEN lines displayed 13 or less mutant types. However, some of the lines (CB3, CB5, CB7, and CB8) displayed more than 50 mutant types. This suggests that target locus cleavage continued in these lines during formation of somatic embryos and/or regeneration of plants since this number is higher than the ploidy level of sugarcane (10–13*x*). Chimerism has also been reported in TALEN and other SSN mediated mutants (Wendt et al. 2013; Feng et al. 2014; Wang et al. 2014; Zhang et al. 2014). Constitutively expressed TALEN or other SSNs have the potential to cleave the target locus before or after the first cell division of somatic embryogenic cells until the binding site is eroded or the spacer region becomes unfavorable for cleavage. As a result, uniform or mosaic individuals can emerge. Mosaic individuals carry different mutant alleles or different types of mutations in the same allele with different frequencies in different tissues.

A positive correlation (R^2^ = 0.85) between lignin reduction and mutations frequencies was observed among the mutant lines. A complete knockout of all homo(eo)logous *COMT* would potentially compromise agronomic performance while maintaining a low frequency of wildtype alleles allows to select events with good agronomic and conversion performance.

Null *COMT brown midrib* mutants in maize (*bm3*) and *Sorghum* (*bmr12*) generally exhibited compromised biomass yields (Miller et al. 1983; Oliver et al. 2005a, b; Sattler et al. 2010). In comparison with null mutants, transgenic plants with RNAi suppression of *COMT* typically showed normal growth performance possibly due to relatively moderate lignin reductions, residual COMT activities, and/or tissue specificity in gene suppression (Pilate et al. 2002; Fu et al. 2011; Jung et al. 2012, 2013; Baxter et al. 2014). Differences in mutation frequencies and lignin reduction among TALEN induced mutant lines in highly polyploid sugarcane should allow the selection of a *COMT* mutant with optimal lignin reduction levels for combining good conversion and agronomic performance.

This opportunity does not exist in diploid species. For example in poplar, a diploid species, CRISPR/Cas9 mediated biallelic *4CL1* mutants showed high uniformity in lignin reduction levels (26 % on average). However no monoallelic *4CL1* mutants were recovered in this study and likely monoallelic mutants would not significantly reduce the lignin content (Zhou et al. 2015). Plant growth performance in *COMT* mutant lines seems to be related with not only lignin reductions but also the tissue culture system. The control and mutants regenerated from callus displayed reduced stem diameter, and further reductions were observed in mutants with 24–32 % lignin reductions. The mutant lines derived from BDE, however, showed comparable stem diameter with control and wild type, even with 22 % lignin reduction. Direct somatic embryogenesis without callus induction potentially minimizes somaclonal variations due to the reduced period of tissue culture (Taparia et al. 2012a, b). In *COMT* RNAi sugarcane plants derived from callus, the line with 12 % lignin reduction displayed improved saccharification efficiency by 32 %, but showed poor agronomic performance (Jung et al. 2013). TALEN induced mutant line derived from BDE, especially CB6 with a higher lignin reduction level (22 %) might display both, superior bioconversion efficiency and agronomic performance.

Brown coloration in leaf midrib and stem has been reported as a typical phenotype observed in *brown midrib COMT* mutants in maize (*bm3*) and *Sorghum* (*bmr12*) (Vignols et al. 1995; Bout and Vermerris 2003). In TALEN induced *COMT* mutants, the lines with relatively low lignin reductions (11–15 % in CB3, CB5, CB7, and CB8) only showed brown coloration in internodes, but not in the leaf midrib. This phenomenon is consistent with RNAi mediated *COMT* suppressed sugarcane with lignin reduction levels ranging from 3.9 to 13.7 % (Jung et al. 2012).

Sugarcane is vegetatively propagated during commercial production by harvesting and planting stem segments. This study confirmed that TALEN mediated *COMT* mutations could be stably transmitted to vegetative progenies. Germline transmission of SSN including TALEN mediated mutation has been demonstrated in a variety of sexually propagated plant species (Feng et al. 2014; Gurushidze et al. 2014; Zhang et al. 2014, 2016; Char et al. 2015). Transferring the *COMT* mutations into other commercial sugarcane cultivars could also be feasible using conventional breeding.

SSN including TALEN can facilitate deregulation processes of genetically engineered plants. Continued expression of SSN is not required once desired modification is induced and selected. The TALEN and selectable marker expression cassette utilized in this study contains loxP sites (Supplemental Fig. 1c). Thus, it is possible to remove TALEN by the Cre/loxP mediated excision process or by segregation after sexual reproduction. Alternatively the genome editing tool could be delivered as a protein to prevent a transgenic footprint (Altpeter et al. 2016).

This study demonstrates that TALEN can be successfully applied for targeted genome editing in sugarcane. TALEN induced *COMT* mutants will be a highly valuable germplasm to enhance the quality of lignocellulosic biomass in sugarcane. Follow-up genotyping will identify individual *COMT* homo(eo)log and/or family members mutated by TALEN and zygosity for each *COMT* homo(eo)log. Field evaluation will elucidate the effect of *COMT* mutations on plant growth performance.

## Materials and methods

### Construction of the TALEN expression vector

TALEN binding and target sites were selected in the conserved region of the first exon among sugarcane *COMT* gene and its putative homo(eo)logs using TALEN^™^ Hit software (http://talen-hit.cellectis-bioresearch.com/). The sequence of sugarcane *COMT* gene (AJ231133) was retrieved from NCBI. Ten tentative consensus (TC) *COMT*s (TC122151, TC126550, TC136346, TC128538, TC129553, TC148041, TC120283, TC129385, TC112705, TC125375) containing the first exon region were retrieved from the sugarcane EST database (DFCI *S.officinarum* Gene Index; http://compbio.dfci. harvard.edu/cgi bin/tgi/geneprod_search.pl). The location and sequence of TALEN target sites, and the corresponding repeat variable di-residues (RVDs) of each TALEN arm were described in Supplemental Fig. 1a, b.

TALEN expression vector was synthesized by GenScript (Piscataway, NJ) carrying two CmYLCV promoters (Stavolone et al. 2003), NtHSP 3′ UTR (GenBank accession no. GU994207), AtHSP 3′ UTR (Nagaya et al. 2010), and loxP sites at both ends of the expression cassette (Supplemental Fig. 1c). TALEN arms were assembled by Cellectis Bioresearch (New Brighton, MN), and left and right TALEN arms were introduced into AscI/PacI and NotI/SbfI cloning sites of the expression vector, respectively. For the selection marker, *npt*II gene cassette under the control of CaMV 35 s promoter, ZmHSP70 intron, and CaMV poly-A signal (Kim et al. 2012) was introduced into the expression vector. The whole expression cassette was then cloned into the binary vector resulting in pTALCOMT for the *Agrobacterium* mediated transformation. Minimum TALEN expression cassette was released by I-SceI digestion, and used for biolistic mediated transformation (Supplemental Fig. 1c).

### Generation of TALEN mutagenized sugarcane

Commercial sugarcane cultivar CP88-1762 was used throughout the study. Immature leaf whorl explants were excised under aseptic conditions from sugarcane tops. TALEN expression cassette was introduced into sugarcane genome through *Agrobacterium* mediated transformation via indirect somatic embryogenesis using callus (AIE) or biolistic mediated transformation via direct somatic embryogenesis (BDE). AIE and BDE were performed following the methods described previously (Taparia et al. 2012a, b; Wu and Altpeter 2015).

Regenerated plants with roots were planted in 0.65 L pots with Fafard No. 2 mix (Sun Gro Horticulture, Apopka, FL) and placed in a growth room with 80 % relative humidity with 16 h photoperiod and 500 µmol m^−2^ s^−1^ light intensity. Plants with three internodes were transplanted to 15 L pots containing Fafard No. 2 mix (Sun Gro Horticulture) and grown to maturity under natural photoperiod in an air-conditioned greenhouse set at 28 °C/22 °C (day/night). Plants were irrigated daily and fertilized biweekly with Miracle-Gro Lawn Food (Scotts Miracle-Gro, Marysville, OH). In each pot, four stalks were maintained by removing juvenile tillers biweekly.

Selected primary mutant lines were clonally propagated using single-node segments of 8-month-old mature plants in 15 L pots containing Fafard No. 2 mix (Sun Gro Horticulture). The growth conditions for vegetative progenies in an air-conditioned greenhouse were the same as described above.

### PCR screening for presence of TALEN in genomic DNA extracts of sugarcane

Genomic DNA was extracted from leaves using DNeasy 96 Plant Kit (Qiagen, Valencia, CA), and 25 ng of DNA was used as template for amplification. TALEN expression cassette specific primers (TALSF and TALSR, Supplemental Table 2) were designed from NtHSP 3′ UTR region, with an expected amplicon size of 332 bp. PCR was performed in the Mastercycler (Eppendorf, Hauppauge, NY) with Hot Start Taq DNA polymerase (NEB, Ipswich, MA) under the following conditions: 95 °C for 30 s denaturation, 35 cycles of amplification at 95 °C for 30 s, 60 °C for 30 s, and 68 °C for 1 min, and final extension at 68 °C for 5 min. PCR products were separated by electrophoresis on 1.0 % agarose gel and visualized after ethidium bromide staining. The lines generating 332 bp PCR product were considered as transgenic lines that displayed a TALEN specific PCR product.

### Capillary electrophoresis

Genomic DNA was extracted from leaves of primary transgenic lines that displayed a TALEN specific PCR product or their vegetative progenies using DNeasy 96 Plant Kit (Qiagen), and 100 ng of DNA was used as a template for PCR amplification. PCR fragment encompassing the TALEN target sites was amplified using 4F and 6-FAM labelled 128R primers (Supplemental Table 2). PCR was performed with Phusion Hot Start II High-Fidelity DNA Polymerase (Thermo Scientific, Waltham, MA) under the following conditions: 98 °C for 30 s, 30 cycles of amplification at 98 °C for 10 s, 68 °C for 15 s, and 72 °C for 15 s, and final extension at 72 °C for 5 min. Capillary electrophoresis of the amplicon was performed by GENEWIZ (South Plainfield, NJ) using Applied Biosystems 3730xl Genetic Analyzers (Life Technologies, Grand Island, NY). Electropherogram was analyzed using the Peak Scanner Software v1.0 (Life Technologies, Grand Island, NY). A peak at 125 bp was considered as wild type *COMT*, and peaks other than 125 bp were analyzed as mutant *COMT*s. Mutation frequency in the *COMT* amplicon population was estimated by quantifying relative fluorescence. Mutation frequency (%) = (sum of peak height of all mutant peaks/sum of peak height of all peaks including wild type peak) × 100.

### Amplicon sequencing

Amplicon sequencing was performed on a 454 sequencing platform. Identical DNA samples and PCR conditions used for CE were employed to prepare amplicon except for using barcoded fusion primers (MIDs and B-key, Supplemental Table 3). The fusion primers consisted of the gene specific sequences tagged with multiplex identifier sequences and sequencing adapters. Amplicon from each sample was purified using Zymoclean Gel DNA Recovery Kit (Zymo Research, Irvine, CA), and quantified using QuantiFluor dsDNA System (Promega, Madison, WI). 400 ng of amplicon from each sample was then pooled and further purified using QIAquick PCR Purification Kit (Qiagen). Sequencing was performed on the GS Junior instrument using Titanium chemistry (454 Life Sciences, Branford, CT) at Eurofins Genomics (Huntsville, AL). Sequence clustering was performed using CD-HIT software v4.5.4, and sequence identity threshold was 100 %.

### Lignin content and composition

Tillers with approximately 30 internodes were harvested from 8-month-old mutant lines, transgenic control for AIE (TCA), transgenic control for BDE (TCB), and wild type plants. Internodes 1–3 below the shoot apical meristem (SAM), all the leaves and leaf sheaths were removed, and internodes 4–12 below SAM were sampled for lignin analysis. The remaining internodes (13–30) were used for vegetative propagation. Total lignin contents were determined using a modified acetyl bromide method as previously described (Jung et al. 2012).

Monolignol compositions were determined using pyrolysis molecular beam mass spectrometer. Each sample was prepared in triplicate by weighing about 1.0–2.5 mg into a stainless metal cup, and pyrolyzed at 500 °C to produce volatile compounds. The volatile compounds were analyzed using the Extrel Core Mass Spectrometers (Extrel CMS, Pittsburgh, PA). NIST 8492 (National Institute of Standards and Technology, lignin content 26.2 %) was used as a lignin standard NIST 8492.

### Analysis of cell wall carbohydrates and soluble solids

Cell wall carbohydrates were analyzed using the National Renewable Energy Laboratory (NREL) protocol by Sluiter et al. (2008). One hundred milligrams of extract-free sample was hydrolyzed with 1 mL of 72 % H_2_SO_4_ at 30 °C for 1 h. Samples were diluted with deionized water to 4 % H_2_SO_4_, and then treated at 121 °C for 1 h. Liberated monomeric sugars were identified and quantified with an Agilent/HP 1200 HPLC equipped with an RI detector (Agilent Technologies, Santa Clara, CA). The HPLC analysis was carried out using a Hi-Plex H column (Agilent Technologies), operating at a flow rate of 0.6 mL min^−1^ using 5 mM H_2_SO_4_ as a mobile phase. A PAL-1 portable refractometer (ATGO U.S.A., Inc., Bellevue, WA) was used to determine the percentage of soluble solids in the extracted juice (˚Brix).

### Statistical analysis

T-test was performed using SAS^™^ v9.3 to determine whether the means of total lignin and monolignol content, cell wall carbohydrates, or stem diameter were statistically significant between the *COMT* mutant lines and wild type plants.

## Electronic supplementary material

Below is the link to the electronic supplementary material.

Supplementary material 1 (DOCX 20 KB)

Supplementary material 2 (PPTX 511 KB)
