# Supplementary material for: TALEN mediated targeted mutagenesis of the caffeic acid O-methyltransferase in highly polyploid sugarcane improves cell wall composition for production of bioethanol
Source: Plant Mol Biol. 2016 Jun 15;92(1):131–42. doi: 10.1007/s11103-016-0499-y (PMC4999463; doi:10.1007/s11103-016-0499-y)
Supplement: Supplementary file 1 — Supplementary material 1 (DOCX 20 KB) [file 11103_2016_499_MOESM1_ESM.docx]

Supplemental Table S1 Monolignol compositions in TALEN induced *COMT* mutants and control plants.

| Lines^1)^ | Monolignol compositions (mg/g DW)^2)^ | | | |
| --- | --- | --- | --- | --- |
|  | H | G | S | S/G |
| WT | 26.9 | 118.3 | 105.0 | 0.89 |
| CA14 | 29.0 | 121.4 | 104.3 | 0.91 |
| CA16 | 19.1 | 112.4 | 48.3* | 0.43* |
| CA17 | 16.4* | 122.4 | 54.6* | 0.45* |
| CB5 | 30.9 | 107.6 | 84.5* | 0.79* |
| CB6 | 21.3 | 105.8 | 70.1* | 0.66* |
| CB7 | 25.2 | 114.8 | 84.1* | 0.73* |

^1)^ Lines include: WT, wild type sugarcanes; CA14, CA16, and CA17, TALEN induced mutant lines generated by AIE; CB5, CB6, and CB7, TALEN induced mutant lines generated by BDE.

^2)^ H: hydroxyphenyl subunit; G: guaiacyl subunit; S: syringyl subunit, DW: dry weight

Asterisk indicates a significant difference in monolignol content between WT and a mutant line (*n*=2, *P*<0.05 in t-test).

Supplemental Table S2 A list of primers used in the study

| Purpose | Primer name | Primer sequence (5′ → 3′) |
| --- | --- | --- |
| TALEN cassette screening | TALSF | AAAGGCGTGTTTGATGTGAA |
|  | TALSR | TCCAAGGACAACTTTAGAAAGAAAA |
| Capillary electrophoresis  of amplicon | 4F | GGCTCGACCGCCGAGGAC |
|  | [6-FAM] 128R | [6-FAM] TCCAGCAGGCCCAGCTCCAG |

Supplemental Table S3 A list of barcoded fusion primers used for amplicon sequencing

| Primer name | Primer sequence (5′ → 3′) |
| --- | --- |
| MID-39_CA1 | CGTATCGCCTCCCTCGCGCCATCAGTACAGATCGTGGCTCGACCGCCGAGGAC |
| MID-30_CA13 | CGTATCGCCTCCCTCGCGCCATCAGAGACTATACTGGCTCGACCGCCGAGGAC |
| MID-05_CA14 | CGTATCGCCTCCCTCGCGCCATCAGATCAGACACGGGCTCGACCGCCGAGGAC |
| MID-28_CA16 | CGTATCGCCTCCCTCGCGCCATCAGACTACTATGTGGCTCGACCGCCGAGGAC |
| MID-50_CA17 | CGTATCGCCTCCCTCGCGCCATCAGACTAGCAGTAGGCTCGACCGCCGAGGAC |
| MID-01_CA25 | CGTATCGCCTCCCTCGCGCCATCAGACGAGTGCGTGGCTCGACCGCCGAGGAC |
| MID-20_CB1 | CGTATCGCCTCCCTCGCGCCATCAGACGACTACAGGGCTCGACCGCCGAGGAC |
| MID-32_CB2 | CGTATCGCCTCCCTCGCGCCATCAGAGTACGCTATGGCTCGACCGCCGAGGAC |
| MID-33_CB3 | CGTATCGCCTCCCTCGCGCCATCAGATAGAGTACTGGCTCGACCGCCGAGGAC |
| MID-46_CB5 | CGTATCGCCTCCCTCGCGCCATCAGTGACGTATGTGGCTCGACCGCCGAGGAC |
| MID-26_CB6 | CGTATCGCCTCCCTCGCGCCATCAGACATACGCGTGGCTCGACCGCCGAGGAC |
| MID-47_CB7 | CGTATCGCCTCCCTCGCGCCATCAGTGTGAGTAGTGGCTCGACCGCCGAGGAC |
| MID-13_CB8 | CGTATCGCCTCCCTCGCGCCATCAGCATAGTAGTGGGCTCGACCGCCGAGGAC |
| B-key | CTATGCGCCTTGCCAGCCCGCTCAGTCCAGCAGGCCCAGCTCCAG |

**Supplemental figure legend**

**Supplemental Fig. S1** TALEN target sites in sugarcane *COMT*, TALEN structure and expression cassette of TALEN and selectable marker gene. (a) The location and sequence of TALEN target sites in sugarcane *COMT* (GenBank accession No. AJ231133). TALEN target site is located in the first exon (exon1) between 52 bp (+52) and 101 bp (+101) downstream of the translation start site. Sequences in red color are left and right TALE binding sites. (b) Schematic diagram of TALEN scaffold and repeat variable di-residues (RVDs) of left and right TALEN. The TALEN has 15.5 repeats, and N-terminal and C-terminal regions are indicated by yellow and green colors, respectively. NLS: Nuclear localization signal. FokI: FokI nucleolytic domain. (c) TALEN and *npt*II expression cassettes in pTALCOMT (10,894 bp). P1: CaMV 35s promoter and ZmHSP70 intron, P2: CmYLCV promoter, T1: CaMV 35s poly-A signal, T2: AtHSP 3′ UTR, T3: NtHSP 3′ UTR. LB: Left border, RB: Right border. I-SceI: I-SceI restriction enzyme sites used to release the minimum expression cassette from pTALCOMT for biolistic mediated gene transfer.

**Supplemental Fig. S2** The yeast single strand annealing assay was used to determine the cleavage rate of the custom synthesized TALEN.

**Supplemental Fig. S3** Frequencies of insertions and deletions (indels) in a total of 381 mutant types identified in the analyzed TALEN induced *COMT* mutant lines. Numbers with or without negative sign in X-axis indicate the size of deletion or insertions.

**Supplemental Fig. S4** Correlation between lignin reduction levels and mutation frequencies among TALEN induced COMT mutant lines with mutation frequencies between 90% and 99%.
