# Supplementary material for: TALEN mediated targeted mutagenesis of the caffeic acid O-methyltransferase in highly polyploid sugarcane improves cell wall composition for production of bioethanol
Source: Plant Mol Biol. 2016 Jun 15;92(1):131–42. doi: 10.1007/s11103-016-0499-y (PMC4999463; doi:10.1007/s11103-016-0499-y)
Supplement: Supplementary file 2 — Supplementary material 2 (PPTX 511 KB) [file 11103_2016_499_MOESM2_ESM.pptx]

## Slide 1
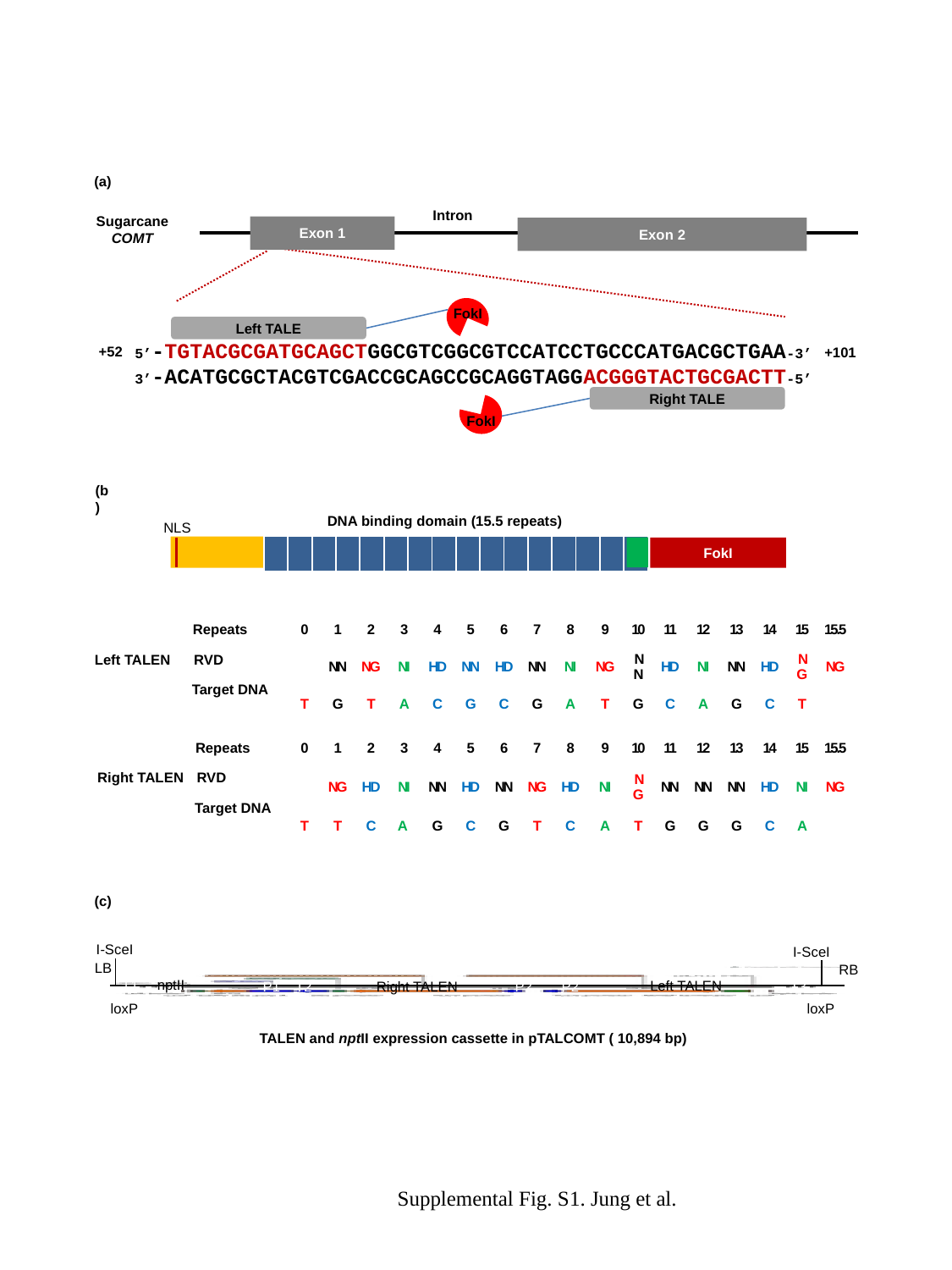

(a)
Sugarcane
COMT
Intron
Exon 1
Exon 2
FokI
Left TALE
5’-TGTACGCGATGCAGCTGGCGTCGGCGTCCATCCTGCCCATGACGCTGAA-3’
3’-ACATGCGCTACGTCGACCGCAGCCGCAGGTAGGACGGGTACTGCGACTT-5’
+52
+101
Right TALE
FokI
(b)
DNA binding domain (15.5 repeats)
NLS
| | | | | | | | | | | | | | | | |
| --- | --- | --- | --- | --- | --- | --- | --- | --- | --- | --- | --- | --- | --- | --- | --- |
FokI
Repeats
| 0 | 1 | 2 | 3 | 4 | 5 | 6 | 7 | 8 | 9 | 10 | 11 | 12 | 13 | 14 | 15 | 15.5 |
| --- | --- | --- | --- | --- | --- | --- | --- | --- | --- | --- | --- | --- | --- | --- | --- | --- |
| | NN | NG | NI | HD | NN | HD | NN | NI | NG | NN | HD | NI | NN | HD | NG | NG |
| T | G | T | A | C | G | C | G | A | T | G | C | A | G | C | T | |
Left TALEN
RVD
Target DNA
Repeats
| 0 | 1 | 2 | 3 | 4 | 5 | 6 | 7 | 8 | 9 | 10 | 11 | 12 | 13 | 14 | 15 | 15.5 |
| --- | --- | --- | --- | --- | --- | --- | --- | --- | --- | --- | --- | --- | --- | --- | --- | --- |
| | NG | HD | NI | NN | HD | NN | NG | HD | NI | NG | NN | NN | NN | HD | NI | NG |
| T | T | C | A | G | C | G | T | C | A | T | G | G | G | C | A | |
Right TALEN
RVD
Target DNA
(c)
I-SceI
I-SceI
LB
RB
P1
Left TALEN
P2
P2
Right TALEN
nptII
T1
T3
T2
loxP
loxP
TALEN and nptII expression cassette in pTALCOMT ( 10,894 bp)
Supplemental Fig. S1. Jung et al.

## Slide 2
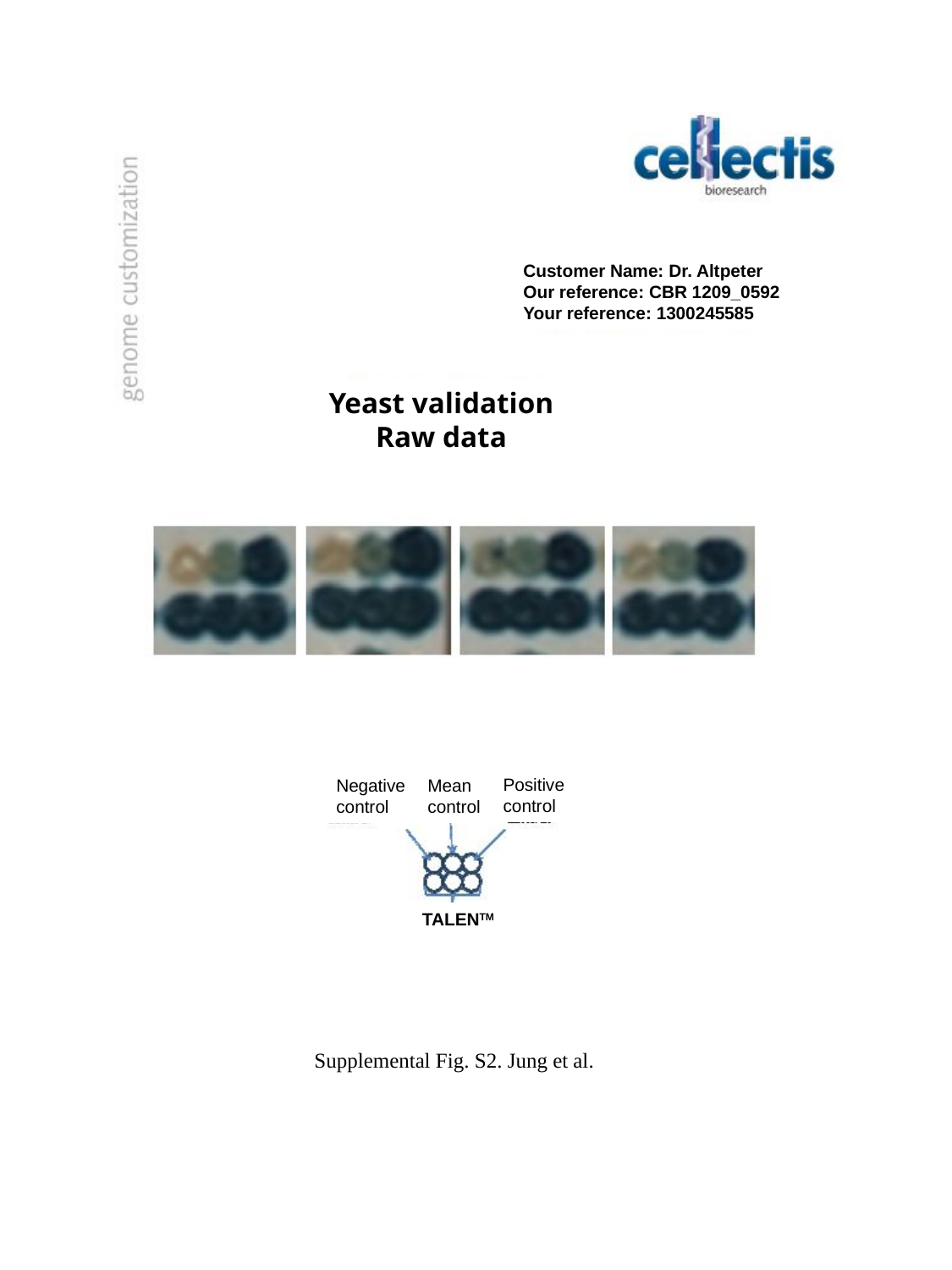

Yeast validation
Raw data
Positive
control
Mean
control
Negative
control
TALENTM
Customer Name: Dr. Altpeter
Our reference: CBR 1209_0592
Your reference: 1300245585
Supplemental Fig. S2. Jung et al.

## Slide 3
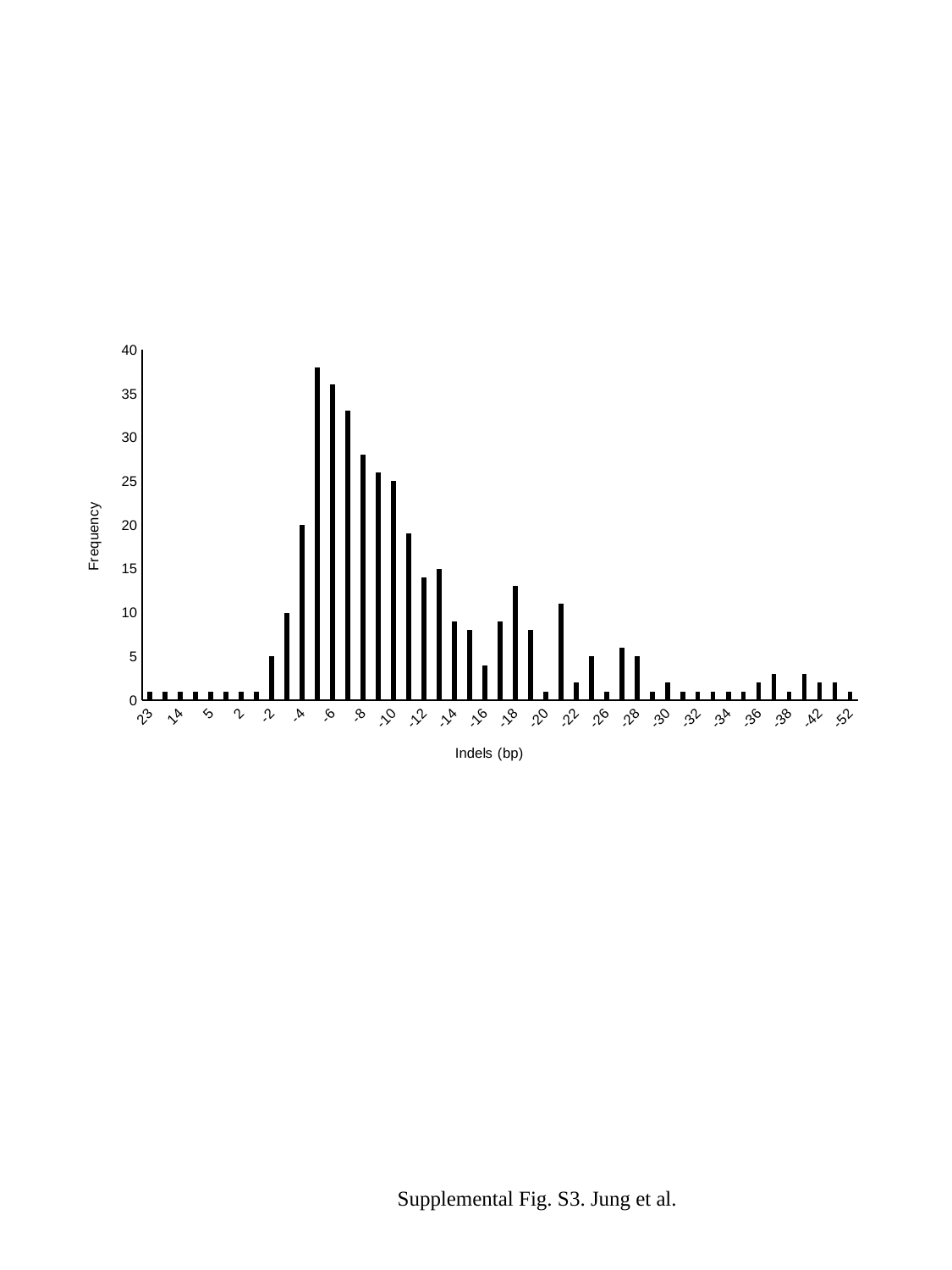

### Chart
| Category | |
|---|---|
| 23 | 1.0 |
| 17 | 1.0 |
| 14 | 1.0 |
| 9 | 1.0 |
| 5 | 1.0 |
| 3 | 1.0 |
| 2 | 1.0 |
| -1 | 1.0 |
| -2 | 5.0 |
| -3 | 10.0 |
| -4 | 20.0 |
| -5 | 38.0 |
| -6 | 36.0 |
| -7 | 33.0 |
| -8 | 28.0 |
| -9 | 26.0 |
| -10 | 25.0 |
| -11 | 19.0 |
| -12 | 14.0 |
| -13 | 15.0 |
| -14 | 9.0 |
| -15 | 8.0 |
| -16 | 4.0 |
| -17 | 9.0 |
| -18 | 13.0 |
| -19 | 8.0 |
| -20 | 1.0 |
| -21 | 11.0 |
| -22 | 2.0 |
| -24 | 5.0 |
| -26 | 1.0 |
| -27 | 6.0 |
| -28 | 5.0 |
| -29 | 1.0 |
| -30 | 2.0 |
| -31 | 1.0 |
| -32 | 1.0 |
| -33 | 1.0 |
| -34 | 1.0 |
| -35 | 1.0 |
| -36 | 2.0 |
| -37 | 3.0 |
| -38 | 1.0 |
| -41 | 3.0 |
| -42 | 2.0 |
| -48 | 2.0 |
| -52 | 1.0 |Supplemental Fig. S3. Jung et al.

## Slide 4
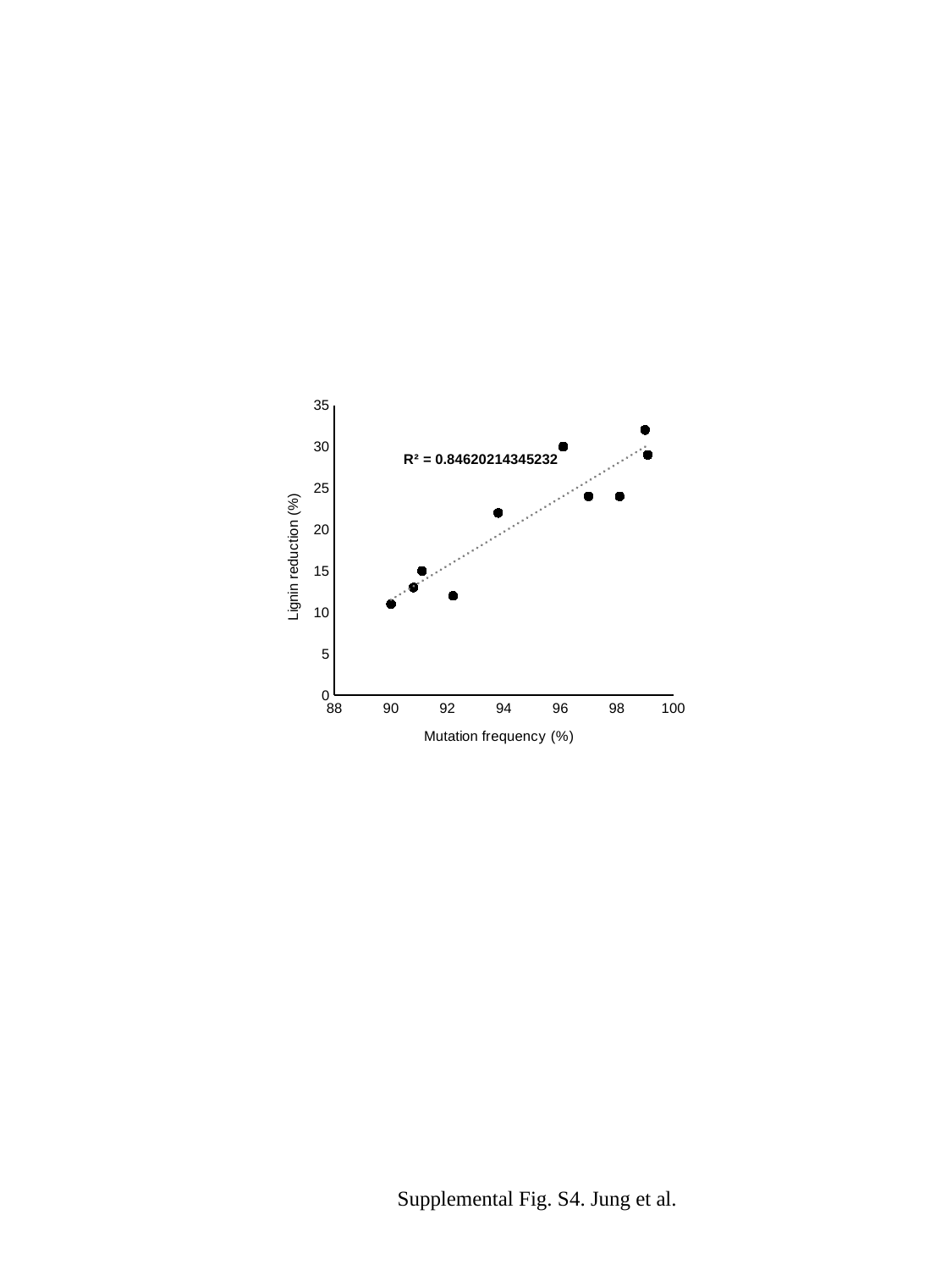

### Chart
| Category | |
|---|---|Supplemental Fig. S4. Jung et al.
